# *In situ* intein-mediated multiprotein assembly via engineered cross-species consortia

**DOI:** 10.3389/fbioe.2025.1529655

**Published:** 2025-04-25

**Authors:** Hao Wang, Jiajia Kang, Hui Gao

**Affiliations:** ^1^ Department of Experiment and Research, South China Hospital, Medical School, Shenzhen University, Shenzhen, China; ^2^ Shenzhen Haolthy Biotechnology Co., Ltd., Shenzhen, China; ^3^ Shenzhen Bay Laboratory, Institute of Molecular Physiology, Shenzhen, China

**Keywords:** protein splicing, *E. coli*, *P. pastoris*, consortia, split intein

## Abstract

Inteins can connect flanking external proteins into a new protein fragment and excise themselves. Here, we report the *in situ* splicing of proteins by engineered microbial consortia. This study pioneers a programmable microbial consortia platform enabling *in situ* protein splicing through split intein-mediated assembly. Engineered *Escherichia coli* with the ePop autolysis system release intein-fused protein fragments via synchronized lysis, while *Pichia pastoris* secretes complementary domains, enabling extracellular reconstitution directly in culture. With the application of integrating quorum-sensing controls and eukaryotic secretion pathways, this approach bypasses *in vitro* purification, supporting scalable one-pot synthesis of multiple functional proteins. The platform’s versatility in logic computation and antibiotic resistance engineering highlights its potential for adaptive biomanufacturing and context-aware biomaterial design.

## Introduction

Split inteins have emerged as potent tools in diverse scientific fields. Their applications range from bi-specific IgG antibody design, antimicrobials, gene therapy, to protein purification, ion channel reconstruction, and non-canonical amino acid incorporation in target proteins, illustrating their broad utility (Wood and Camarero, 2014; Truong et al., 2015; López-Igual et al., 2019; Han et al., 2017; Choi et al., 2018; Bäckhed et al., 2015). Notably, split inteins enable spontaneous self-assembly and protein splicing. They facilitate the reconstitution of coding sequences with minimal impact on the original protein’s function due to the negligible “scarring” effect (Belfort et al., 2006). Nonetheless, implementing split inteins requires junction sequences near insertion sites, a challenge addressable by identifying a broader range of intein species compatible with target proteins. Moreover, the discovery of orthogonal intein libraries has expanded possibilities for simultaneous assembly of peptides or proteins (Pinto et al., 2020).

Traditional protein splicing, confined to *in vivo* and *in vitro* reactions, involved separate expression of chimeric proteins, followed by cell lysis and purification, and finally, splicing induction via DTT (Dithiothreitol) and pH adjustments (Pinto et al., 2020; Wang et al., 2022a; Wang et al., 2018). However, this method is impractical for large-scale manufacturing due to its labor-intensive nature. Synthetic biologists have recognized the importance of co-culture techniques, especially for cell consortia systems, with their vast potential in research and industrial applications. Co-culture techniques are greatly important to synthetic biologists, for cell consortia systems provide an enormous potential for research as well as industrial applications, with many proof-of-concept studies being carried out nowadays (Dai et al., 2019; Wang et al., 2022b). We tried to express two separate parts of one protein in one cultural system and find out whether they could be assembled upon intein splicing directly in the culture medium. Our approach aimed to express two protein segments in a single culture, examining their direct assembly via intein splicing in the medium. This led to the development of an *in situ* splicing method, enabling efficient soluble expression and splicing in a one-pot process. We integrated an engineered bacteria cell lysis system, the ePop system, to facilitate automatic cell lysis and protein splicing (Dai et al., 2019). The ePop plasmid is a genetic engineering tool designed for synchronized bacterial lysis in co-culture systems, leveraging quorum-sensing mechanisms and toxin-antitoxin principles (Dai et al., 2019). Also, co-culturing *Pichia pastoris*, a versatile yeast known for efficient secretion of recombinant proteins, with this auto-cell lysis system successfully yielded spliced proteins via split intein mediation (Li et al., 2015). This breakthrough establishes a cross-species consortia system for future diverse protein assembly applications.

## Results

### Schematic representation of *in situ* protein splicing as well as its potential applications across diverse fields

**FIGURE 1:**
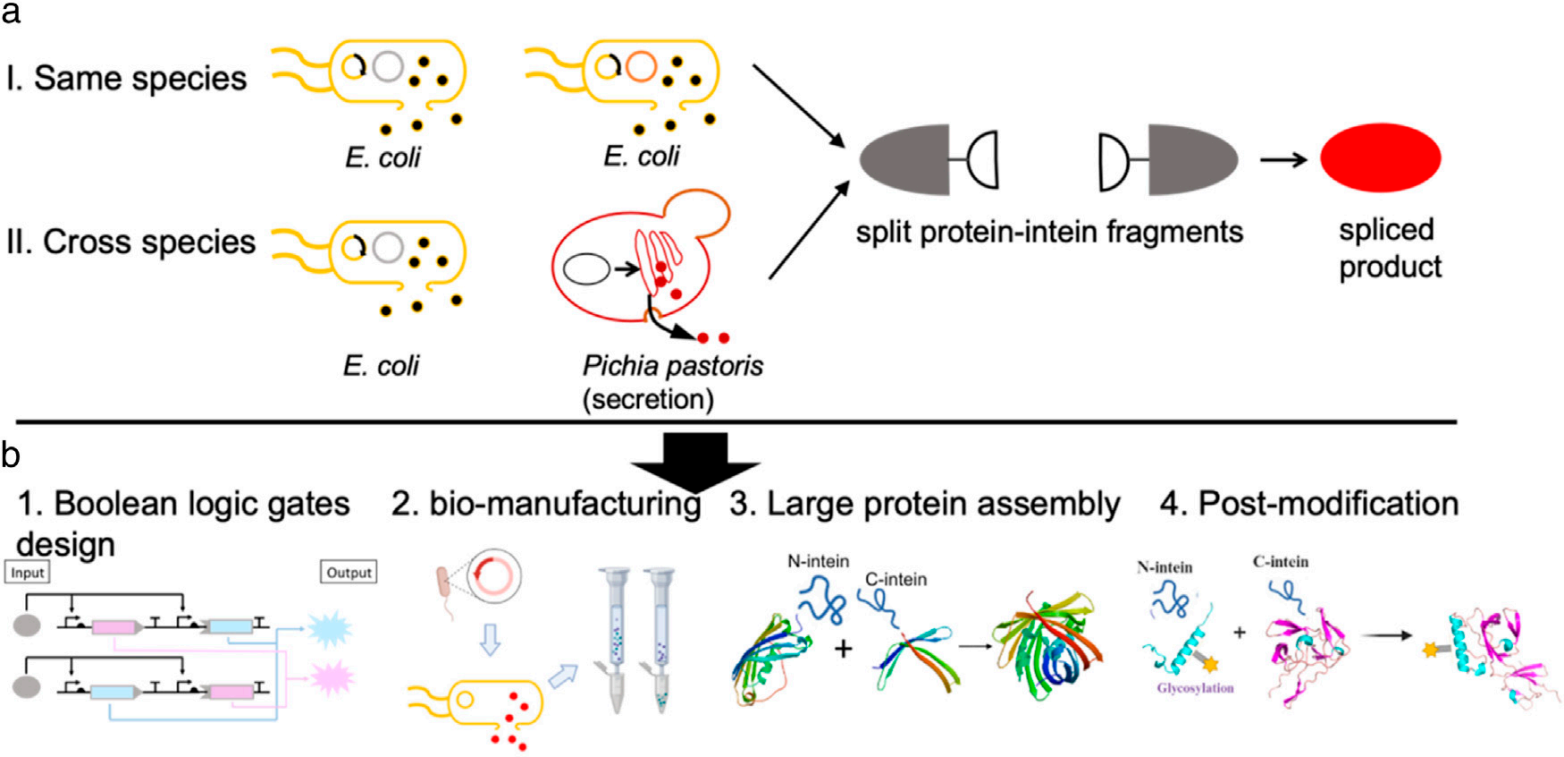
*In situ* protein splicing by engineered consortia. **(a)** Schematic representation of *in situ* protein splicing by either *E. coli* culture or *E. coli-P. pastoris* consortia to assemble red florescent protein (mCherry). **(b)** *In situ* protein splicing can be applied in different research aspects such as Boolean logic gate design, simplifying bio-manufacturing processes, facilitating large protein assembly, enhancing protein post-modification as explained above.

This study presents a design aimed at automating the protein splicing process. In this design, split intein chimeric proteins were expressed in either an *E. coli* or an *E. coli-P. pastoris* co-culture environment, with protein splicing triggered upon the release of both chimeric proteins under optimal chemical conditions (Pinto et al., 2020; Wang et al., 2018). The *in situ* protein splicing method developed here holds significant potential for application in Boolean logic gate design, simplifying bio-manufacturing processes, facilitating large protein assembly, enhancing protein post-modification, and more (Figure 1).

### Designing the co-culture scheme for *in situ* protein splicing of extein-split intein chimeric proteins

Two previously reported split inteins species, *Ssp* GyrB (G) and gp418 (g) were applied in this study (Pinto et al., 2020). Characterization of the splicing efficiency of *Ssp* GyrB and gp418 was first examined *in vivo* in *E. coli* by co-transforming two plasmids expressing mCherry^N^-GyrB^N^, GyrB^C^-mCherry^C^ chimeric proteins and mCherry^N^-gp418^N^, gp418^C^-mCherry^C^ respectively. The split sites in mCherry for both inteins (GyrB and gp418) are located at D154-G155, a position previously reported to allow full recovery of fluorescence activity when reconstituted (Pinto et al., 2020). This split site applies to all contexts involving mCherry mentioned in the text unless otherwise specified. The splicing rate of the two split inteins was measured using a microplate reader (Biotek Ltd.), as only the spliced mCherry exhibited high fluorescent signals, while the two split halves did not produce any detectable fluorescent signals. Cell cultures showed 1,200–2,000 a.u. (arbitrary units) at 580 nm excitation-610 nm emission, followed by native PAGE analysis, exhibiting good performance on protein splicing (Supplemental Figure 1).

Also, we transformed the BL21 (DE3) cells with the ePop plasmid. The ePop system, a sensitive tool for auto cell lysis, senses cell density and triggers lysis under high-density conditions (Dai et al., 2019). We co-transformed *E. coli* cells with the extein-split intein fusion constructs (in the low copy number plasmid p15A), and the high copy number ePop plasmid. Upon IPTG (Isopropyl β-D-1-thiogalactopyranoside) induction, both the extein^N^-GyrB^N^- and GyrB^C^-extein^C^ chimeric peptides were expressed and released into the culture medium when ePop triggered cell lysis, enabling direct interaction and functional protein splicing by split intein.

### *In situ* protein splicing of mCherry-GyrB fusion via co-culture systems

**FIGURE 2:**
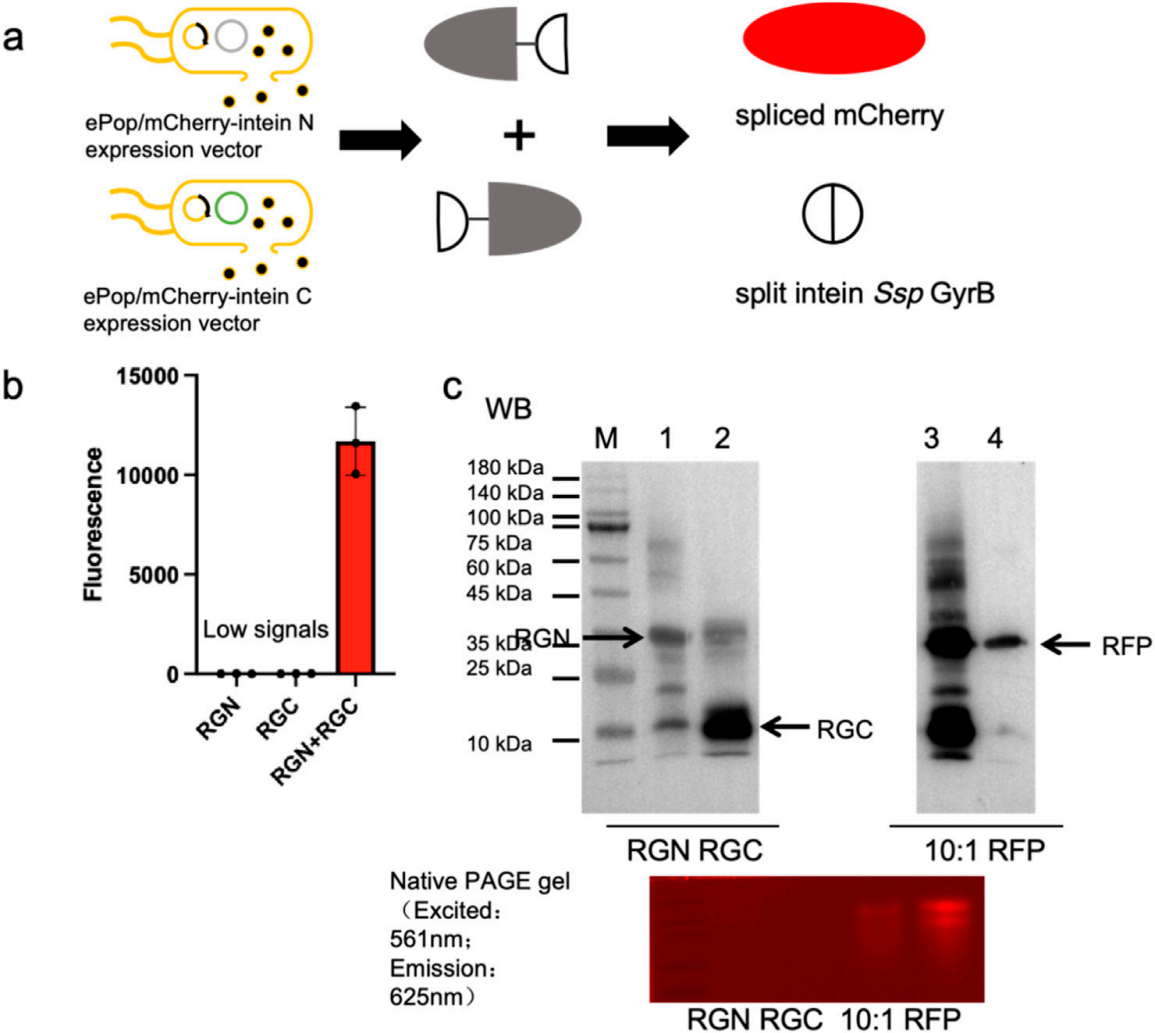
Demonstration of *in situ* protein splicing by *E. coli* culture to assemble mCherry. (a) Schematic representation of *in situ* protein splicing by *E. coli* culture to assemble mCherry. (b) Eluted samples were loaded in 384-microplates and analyzed by Biotek microreader at 580 nm excitation-610 nm emission. (c) Western blots and Native PAGE analysis results of eluted samples of extein^N^-GyrB^N^- as well as GyrB^C^-extein^C^ chimeric proteins, spliced RFP (mCherry) and RFP positive control. 10:1: The maximum mCherry splicing rate was achieved when the subculture ratio of cells expressing the split mCherry^N^ lobe-split intein chimeric protein to the split mCherry^C^ lobe-split intein chimeric protein is 10:1. The extein^N^-GyrB^N^ chimeric protein was engineered with a N-terminal His-tagged and the GyrB^C^-extein^C^ chimeric protein was engineered with a C-terminal His-tagged. The mCherry protein was engineered with a C-terminal His-tagged, enabling Western blot detection via Anti-His antibody (clone HIS.H8, Mouse IgG1κ, Abcam ab18184) at 1:5,000 dilution in 5% BSA-TBST.

**FIGURE 3 F3:**
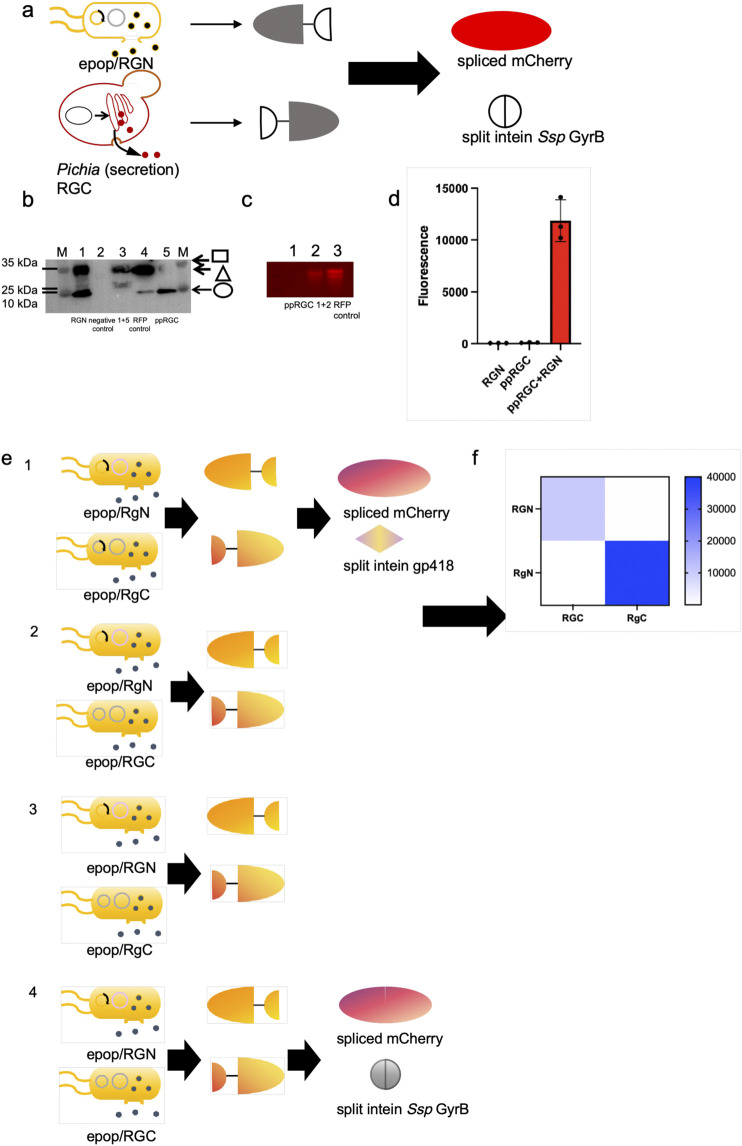
Demonstration of *in situ* protein splicing by *E. coli-P. pastoris* consortia to assemble mCherry and orthogonal test through mCherry splicing. (a) Schematic representation of *in situ* protein splicing by *E. coli-P. pastoris* consortia to assemble mCherry. (b, c) Western blots and Native PAGE analysis results of eluted samples of *E. coli* expressed extein^N^-GyrB^N^- (Square) as well as *P. pastoris* secreted GyrB^C^-extein^C^ (Circle) chimeric proteins, spliced RFP (mCherry) (Triangle) and RFP positive control. The mCherry protein was engineered with a C-terminal His-tagged, enabling Western blot detection via Anti-His antibody (clone HIS.H8, Mouse IgG1κ, Abcam ab18184) at 1:5,000 dilution in 5% BSA-TBST. 1+5 and 1+2: Detection of purified proteins from co-cultured samples of *E. coli* expressing RGN and *Pichia pastoris* expressing ppRGC. The extein^N^-GyrB^N^ chimeric protein was engineered with a N-terminal His-tagged and the GyrB^C^-extein^C^ chimeric protein was engineered with a C-terminal His-tagged. (d) Eluted samples were loaded in 384-microplates and analyzed by Biotek microreaderat 580 nm excitation-610 nm emission. (e, f) Orthogonality of the two selected split intein pairs (*Ssp* GyrB and gp418 inteins) were tested by measuring the fluorescence of *E. coli* cells co-expressing 4 combinations of each N- and C-terminal chimeric protein halves (2 N × 2 C). In this matrix, each split intein has one non-cognate combination and their fluorescence values were normalized to that of the cognate pair.

We explored protein splicing in a cell culture environment. Two *E. coli* strains, one expressing mCherry^N^-GyrB^N^- (RGN) and the other GyrB^C^-mCherry^C^ (RGC), were co-cultured in M9 medium and induced overnight (Figure 2a). The ePop auto-lysis plasmid facilitated cell lysis at high cell densities, releasing chimeric proteins into the environment. Additionally, we tested cross-species cultivation with *Pichia Pastoris*, expressing mCherry^N^-GyrB^N^- (RGN) in *E. coli* BL21 (DE3) (equipped with ePop) and GyrB^C^-mCherry^C^ (expressing RGC from *P. Pastoris*, named ppRGC in the text) in *P. Pastoris* X-33 (Figure 3a). Both experiments confirmed that intein-mediated protein splicing occurred directly in cell cultures. Splicing was induced by 4 mM DTT (Dithiothreitol) and pH 9.0, with successful reactions indicated by strong fluorescent signals at specific wavelengths, further validated by Western blot and native SDS-PAGE (Figures 2b,c, 3b–d [Fig F3]).

### *In situ* same strains co-culture for characterization of bio-orthogonality of the two selected split intein species

To determine whether two selected split intein pairs (*Ssp* GyrB and gp418 inteins) could be used simultaneously in the same application, we tested their orthogonality by measuring the fluorescence of *E. coli* cells co-expressing 4 combinations of each N- and C-terminal chimeric protein halves (2 N × 2 C). In this matrix, each split intein has one non-cognate combination and their fluorescence values were normalized to that of the cognate pair. The result showed that the values of the fluorescent signals generated by non-cognate combinations were significantly lower than those from the cognate pairs, and exhibited good orthogonality of the two selected intein species (Figures 3e,f).

### Concurrent *in situ* splicing of mCherry and β-lactamase in same strain co-cultures

**FIGURE 4:**
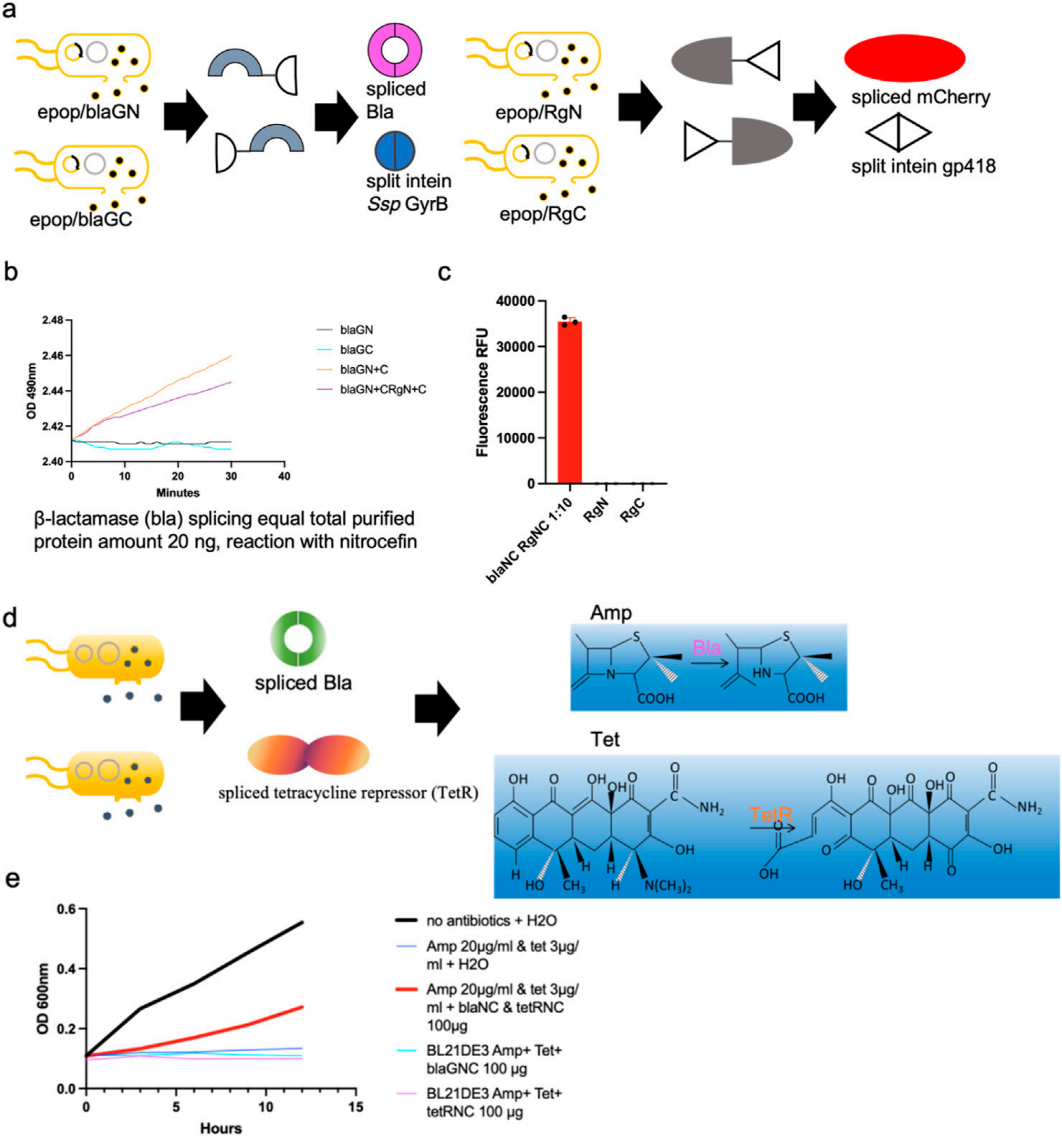
Applications of the *in situ* splicing method. **(a)** Schematic representation of concurrent *in situ* splicing of mCherry and β-Lactamase in same strain co-cultures. **(b, c)** Purified protein mixtures exhibit around 35,000 a.u. at 580 nm excitation-610 nm emission and reacted with nitrocefin substrates to increase absorbance values at OD490nm, showing both mCherry and β-lactamase were able to splice accordingly after induction of splicing reaction. 1:10: Cells harboring vectors for His-tagged C-lobe-split intein chimeric proteins and ePop autolysis plasmids were inoculated into 100 mL M9 medium supplemented with 100 μg/mL kanamycin and 100 μg/mL chloramphenicol at a 1:10 inoculation ratio. Similarly, cells expressing His-tagged N-lobe-split intein chimeric proteins with ePop plasmids were cultured in 100 mL identically supplemented M9 medium using a 1:10 inoculation ratio **(d)** Schematic representation of expressing four chimeric proteins in a single system in order to synthesize both spliced β-lactamase and tetracycline. **(e)** Splicing was induced and observed growth in *E. coli* cells only in the presence of all four proteins, demonstrating the feasibility of the *in situ* splicing approach.

We next applied the *in situ* splicing system to co-express multiple chimeric peptides and splice simultaneously. The mCherry^N^-gp418^N^ (RgN), gp418^C^-mCherry^C^ (RgC), Bla^N^-GyrB^N^ (BlaGN) and GyrB^C^-Bla^C^ (BlaGC) were expressed and released to the M9 culture medium. The split site in β-Lactamase for the GyrB intein is located at G172-E173, a position previously reported to allow full recovery of enzymatic activity when reconstituted (Pinto et al., 2020). This split site applies to all contexts involving β-Lactamase mentioned in the text unless otherwise specified. Purified protein mixtures exhibit around 35,000 a.u. (arbitrary units) at 580 nm excitation-610 nm emission and reacted with nitrocefin substrates to increase absorbance values at OD490nm, showing both mCherry and β-lactamase were able to splice accordingly after induction of splicing reaction (Figures 4a–c; Supplemental Figure 4). Nitrocefin is a chromogenic substrate that undergoes a color change upon cleavage by β-lactamase, providing a visible indicator of enzyme activity. The absorbance of the cleaved product is typically measured at a wavelength of 490 nm.

### Application in co-culture systems for protein splicing of antibiotic repressors

The *in situ* splicing system was further tested for the production of two antibiotic resistance proteins, β-lactamase and tetracycline resistance protein. We wanted to develop a practical way to reduce the overuse of antibiotics causing environmental problems in water. We applied *in situ* splicing to produce antibiotic-resistant proteins by expressing four chimeric peptides within a single system: Bla^N^-GyrB^N^, GyrB^C^-Bla^C^, Tet^N^-M86^N^, and M86^C^-Tet^C^. M86, an intein species selected from a previous report, was used due to its strong splicing activity (Pinto et al., 2020). As for the split site in the tetracycline resistant protein, it is located at L70-E71 for the M86 intein, a position previously reported to allow full recovery of enzymatic activity when reconstituted (Pinto et al., 2020). We induced splicing and observed growth of *E. coli* cells only in the presence of all four proteins, demonstrating the feasibility of this approach (Figures 4d,e).

### Boolean logic gates circuits via *in situ* splicing systems

**FIGURE 5:**
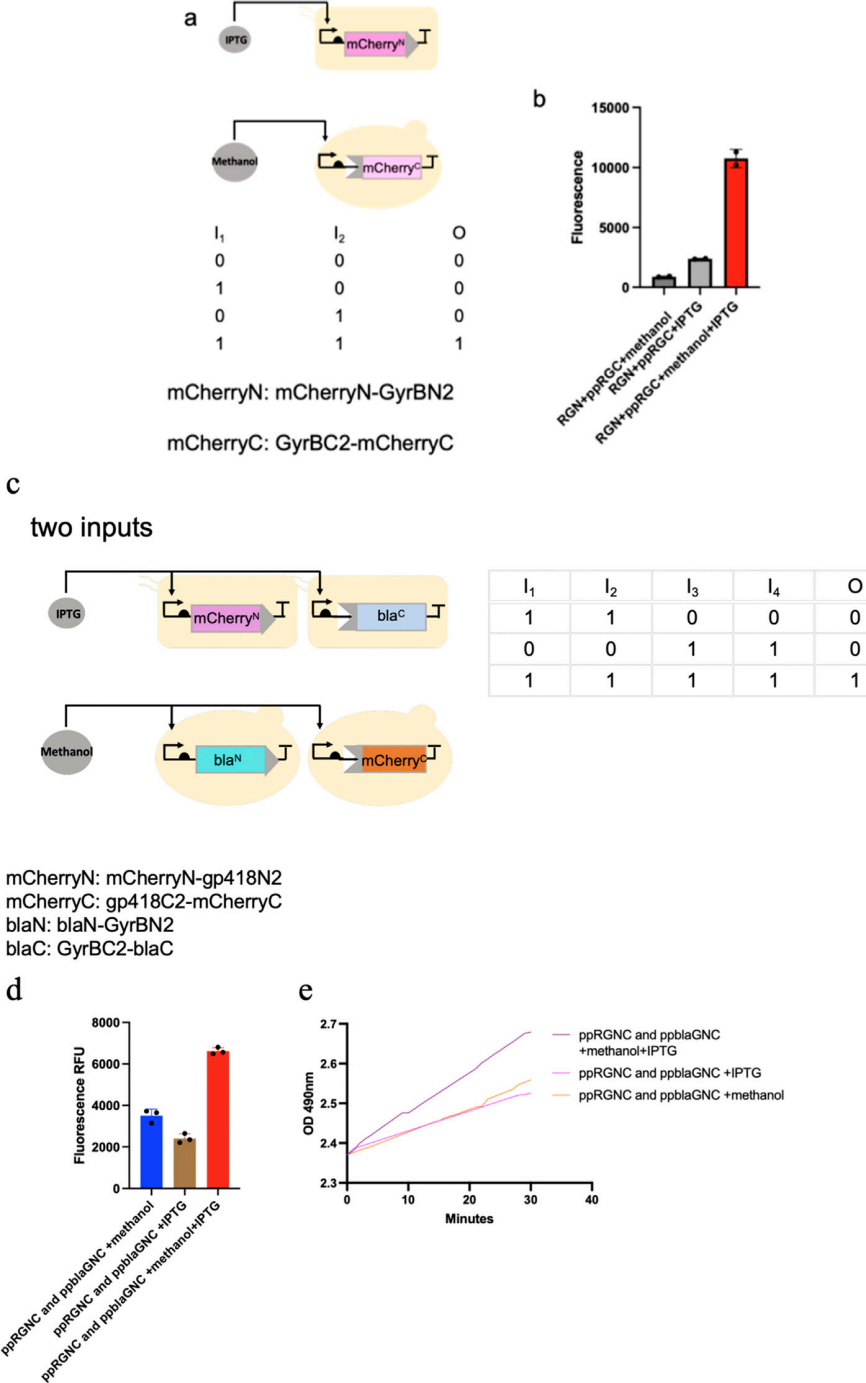
Presentation of logic AND gates through the *in situ* splicing method. **(a–e)** one-input and two-input logic AND gates were constructed, controlling either split mCherry-intein halves or both split mCherry-intein halves and split β-Lactamase-intein halves with IPTG and methanol in co-culture. The mCherry^N^ and β-Lactamase^C^ lobe were released from the *E. coli* host to the culture medium, while the mCherry^C^ and β-Lactamase^N^ lobe were secreted using an α-factor signal peptide from *P. pastoris*, and the splicing reaction of the split chimeric proteins was induced. The system showed fluorescent signals only with both inducers present.

We constructed two-input logic AND gates, controlling split mCherry-intein halves with IPTG (Isopropyl β-D-1-thiogalactopyranoside) and methanol in co-culture. The mCherry^N^ lobe was released from the *E. coli* host to the culture medium while the mCherry^C^ lobe was secreted using an α-factor signal peptide from *P. pastoris*, and the splicing reaction of split chimeric proteins was induced. The system showed fluorescent signals only with both inducers present (Figures 5a,b).

We also built a system where both mCherry and β-lactamase were spliced under dual inducer conditions. We built this system where the mCherry^N^-gp418^N^ and GyrB^C^-bla^C^ were expressed in *E. coli*, while the gp418^C^-mCherry^C^ and Bla^N^-GyrB^N^ (expressing Bla^N^-GyrB^N^ from *P. Pastoris*, named ppblaGN in the text) were expressed in *P. pastoris*. *Fluor*escent signals and absorbance measurements for nitrocefin reactions reached peak values under combined IPTG and methanol induction, whereas both parameters remained low when either inducer was applied alone (Figures 5c–e).

## Discussion

In recent years, with the rise and development of synthetic biology, many novel molecular biological tools have been thoroughly researched and innovated. There is an increasing number of reports on the use of the concept of co-culturing strains for achieving effective division of labor among components and efficiently produce active ingredients (Dai et al., 2019; Wang et al., 2022b; Zhu et al., 2024); however, there have been no reported cases of using co-culturing of microbial consortia for protein assembly. Here, we have successfully implemented and experimentally validated the original design of using co-culturing of strains to efficiently assemble active proteins using split intein for the first time. This *in situ* assembly system integrates two complementary strategies: (1) leveraging the ePop bacterial lysis element to induce controlled autolysis of *E. coli* within the culture medium, enabling extracellular release of recombinant proteins, while simultaneously exploiting *P. pastoris*’s eukaryotic secretory machinery to enhance solubility and yield of challenging proteins/peptides; and (2) employing split intein-mediated segmental expression for environmental self-assembly of pre-functional protein fragments into fully active conformations. This application has significant implications for increasing the gene loading capacity, protein solubility, and activity enhancement, as well as for the improvement of recombinant protein production processes. Moreover, whether it's consortia of the same microbial community or cross-species co-culturing systems, we have demonstrated that *in situ* assembly can assemble two types of active proteins in the same culture environment simultaneously with the use of bio-orthogonal split intein tools. Based on this theoretical foundation, we have successfully achieved the simultaneous production of two antibiotic resistance proteins, Bla and TetR, and the design of a logic gate pathway controlled by two inducers. Lack of methods to precisely control cell growth rates between *E. coli* and *P. pastoris* has been addressed previously (Wang et al., 2022b), which could significantly affect both the communication and the division of labor between the sub-populations. However, in our case, a cross-species co-culture system with *E. coli* and *P. pastoris* was established, which maintains viability in a 1:1 hybrid of M9 and BMMY media–a composition balancing the nutritional requirements of both species. Experimental validation using a 1:1 initial inoculation ratio demonstrated efficient intein-mediated splicing of split mCherry, as evidenced by robust red fluorescence and Western blot detection of full-length RFP. The near-complete reaction between N-terminal and C-terminal fragments confirmed precise intein reconstitution, with the reassembled protein retaining native fluorescence properties. This hybrid medium strategy successfully supported interspecies compatibility while preserving the structural integrity and functionality of the spliced fluorescent protein. While the aforementioned issue does not compromise protein splicing success in our *in situ E. coli-P. pastoris* co-culture system, we are actively pursuing strategies to enhance splicing efficiency by resolving interspecies growth rate disparities through innovative consortium engineering.

Split proteins via intein splicing offer critical advantages by enabling programmable control over protein activity, particularly in contexts like antimicrobial peptide production, where basal expression poses a significant barrier. Split inteins provide a fundamentally different layer of precision by decoupling protein assembly from transcription/translation, thereby mitigating unintended basal activity and enabling multi-input logic gating. For example, antimicrobial peptides exhibit inherent toxicity to host cells, and even low-level “leaky” expression during fermentation can impair cell viability or necessitate costly purification steps to remove inactive precursors (López-Igual et al., 2019). Split inteins circumvent this by dividing the peptide into two non-functional fragments that only reconstitute into an active form upon intein-mediated trans-splicing, which can be tightly regulated by external inducers such as light, temperature, or small molecules. This modularity allows the design of AND gates where the functional peptide is produced exclusively when two independent promoters (e.g., responsive to different inducers) are activated, effectively eliminating background activity (López-Igual et al., 2019; Pinto et al., 2020; Ho et al., 2021).

Moving forward, a considerable array of potent medications, including antibody-drug conjugates (ADCs), bi-specific antibodies (BsAbs), and Fc-fusion proteins (Fc: fragment crystallizable), among others, will play a pivotal role in the field of human health (Akiba et al., 2021; Erlandson et al., 2022; Sassoon and Blanc, 2013). These are the products of fusion gene expression, identified for their high specificity and requiring extensive fusion protein expression and activity assessment. Future demands for constructing numerous fusion proteins to screen drug activity will render traditional methods—which require laborious individual gene assembly for each protein—prohibitively inefficient (Kruse et al., 2008). The escalating demand for diverse fusion protein constructs inevitably leads to combinatorial expansion of required expression vectors under conventional approaches. Split intein-mediated assembly fundamentally alters this paradigm by decoupling vector construction from target complexity—total vectors scale linearly with the number of chimeric elements (peptides/protein domains) rather than exponentially with fusion permutations (Elleuche and Pöggeler, 2010). This orthogonal assembly strategy synergizes with ePop-enabled coculture systems, where engineered microbial consortia achieve coordinated expression and autonomous protein splicing through interspecies metabolic complementarity and controlled lysis (Warren et al., 2013). Such integration paves the way for next-generation activity detection platforms, particularly when augmented by AI-driven high-throughput screening systems that automate: (1) Real-time optimization of intein splicing efficiency via machine learning analysis of fluorescence/absorbance kinetics (Listwan et al., 2009); (2) Predictive modeling of orthogonal intein pair compatibility to minimize cross-reactivity (Elleuche and Pöggeler, 2010); (3) Robotic handling of co-culture parameter spaces (induction timing, species ratios, medium composition) to maximize functional protein yields (Steinmetz and Auldridge, 2017; Olszakier et al., 2023).

## Materials and methods

### Strains and standard growth conditions

Plasmid cloning work was all performed in *E. coli* DH5a and Top10 strains (Transgene *Inc.*). *E. coli* BL21 (DE3) (Transgene *Inc.*) was applied for protein expressions as indicated. For transformation and plasmid extraction, *E. coli* DH5a and Top10 cells were cultivated in LB (Lysogeny Broth) medium (10 g L^−1^ tryptone, 5 g L^−1^ yeast extract, 5 g L^−1^ NaCl). *Pichia pastoris* X-33 was cultivated in YPD (Yeast Extract Peptone Dextrose, 10 g L^−1^ yeast extract, 20 g L^−1^ peptone, 100 g L^−1^ dextrose) for plasmid transformation.

For protein expression, *E. coli* BL21 (DE3) cells were first inoculated in LB (Lysogeny Broth) medium containing 2% glucose for overnight growth, and subcultured into M9 (Sangon Biotech Co., *Ltd.*) medium on the next day. Antibiotics were used when required at final concentrations of 100 μg mL-1 of kanamycin, chloramphenicol and zerocin. *Pichia pastoris* X-33 was inoculated in BMGY (10 g L^−1^ yeast extract, 20 g L^−1^ peptone, 100 mM potassium phosphate, 1.34% YNB, 4% × 10%^−5^% biotin, 1% Glycerol) medium first for overnight growth, and subcultured into BMMY (10 g L^−1^ yeast extract, 20 g L^−1^ peptone, 100 mM potassium phosphate, 1.34% YNB, 4% × 10%^−5^% biotin, 0.5% methanol) medium for protein expression for 3 days induced by addition of methanol (working concentration of methanol equals to 0.5%) per day.

### Materials, preparation, and assembly of DNA constructs

LB and M9 media were purchased from Sangon Biotech (Shanghai, China). Anti-His antibody for Western blotting was obtained from Abcam (clone HIS.H8, Mouse IgG1κ, Abcam ab18184, Cambridge, United Kingdom). Nitrocefin, a chromogenic β-lactamase substrate, and tetracycline were acquired from Sigma-Aldrich (St. Louis, MO, United States). Antibiotics (kanamycin, chloramphenicol, ampicillin) and D-glucose were sourced from Sangon Biotech.

The target DNA fragments were PCR-amplified using primers containing 15-bp homology arms. Subsequently, these fragments were directionally cloned into destination vectors through homologous recombination-mediated assembly (C112 kit, Vazyme Biotech). For quality control assurance, all plasmid constructs and oligonucleotides were commercially synthesized by General Biologicals Corporation (Anhui, China), with sequence-verified clones being utilized for downstream applications.

### Bacterial and fungi transformations for *in situ* assays

For single plasmid transformation, 10–50 ng of the plasmid were incubated in a 1 mL centrifuge tube, supplemented with 200 μL competent cell (Transgene *Inc.*) and incubated on ice for 10 min before electroporation in a cuvette at 1.8 V. For double plasmids transformation, 10–50 ng of each plasmid were incubated in a 1 mL centrifuge tube, supplemented with 200 μL competent cell (Transgene *Inc.*) and incubated on ice for 10 min before electroporation in a 1 mm cuvette at 1.8 V. Cells were then diluted with 500 μL LB and incubated in a shaker at 37°C, 220 rpm for 30 min before spread on agar plates with both 100 μg mL^−1^ of kanamycin and chloramphenicol supplemented.

For transformation and integration of DNA fragments into *P. pastoris* X-33 genome, 200 ng of the plasmid containing the AOX expression cassette were first digested with Sa*cI* enzyme for plasmid linearization. Then, linearized plasmids were purified and transformed into freshly grown *P. pastoris* X-33 cells at OD600 nm = 0.6–0.8 by electroporation in a 2 mm cuvette at 3 V. Cells were then diluted with 300 μL YPD and 300 μL 1 M sorbitol, followed by incubation in a shaker at 30°C, 220 rpm for 30 min before spreading them on agar plates, both supplemented with zeocin to a final concentration of 250 μg mL^−1^.

### *In situ* two strains co-culture for split mCherry-split intein chimeric protein splicing

For the characterization of *in situ* protein splicing in a co-culture of 2 *E. coli* strains, 10 mL of LB medium enriched with 2% glucose and supplemented with 100 μg/mL each of kanamycin and chloramphenicol were used to inoculate two sets of *E. coli* BL21 (DE3) cells. One set carried vectors for expressing his-tagged split mCherry^N^ lobe-split intein chimeric proteins and ePop autolysis plasmids, while the other expressed his-tagged split mCherry^C^ lobe-split intein chimeric proteins and ePop autolysis plasmids. The cells were incubated at 37°C and 220 rpm overnight. The cells carrying vectors expressing the his-tagged split mCherry^N^ lobe-split intein chimeric proteins and the ePop autolysis plasmids were then subcultured into 10 mL M9 medium with both 100 μg mL^−1^ of kanamycin and chloramphenicol supplemented following a 1:10 subculture ratio, and the other cells carrying vectors expressing the his-tagged split mCherry^C^ lobe-split intein chimeric proteins and the ePop autolysis plasmid were subcultured into 10 mL M9 medium with both 100 μg mL^−1^ of kanamycin and chloramphenicol supplemented following a 1:100 subculture ratio. Protein expression was induced by a final concentration of 0.1 mM IPTG (Sangon Biotech Co., *Ltd.*) and growing at 37°C, 220 rpm for 24 h. The maximum mCherry splicing rate was achieved when the subculture ratio of cells expressing the split mCherry^N^ lobe-split intein chimeric protein to the split mCherry^C^ lobe-split intein chimeric protein is 10:1.

For cross-species co-cultivation, *P. pastoris* strain X33 harboring the pICZ-RGC plasmid (expressing RGC from *P. Pastoris*, named ppRGC in the text) was inoculated into 10 mL BMGY medium (10 g/L yeast extract, 20 g/L peptone, 100 mM potassium phosphate, 1.34% yeast nitrogen base [YNB], 4 × 10^−5^% biotin, 1% glycerol) and incubated overnight at 30°C with 220 rpm shaking to reach mid-log phase (OD_600_ ≈ 6.0). Concurrently, *E. coli* BL21 (DE3) cells carrying the p15A-RGN plasmid were cultured in 10 mL LB medium supplemented with 2% glucose, 100 μg/mL kanamycin, and 100 μg/mL chloramphenicol. After 16-h pre-cultivation, both cultures were subcultured at a 1:10 ratio into a 100 mL mixed medium comprising 50 mL LB and 50 mL BMMY supplemented with 0.2 mM IPTG (identical to BMGY but substituting glycerol with 0.5% methanol as the carbon source). The co-culture system was maintained at 30°C with 220 rpm agitation for 72 h, with daily methanol supplementation to sustain a final concentration of 0.5% (v/v), thereby synchronizing protein induction phases between the two species.

Cell lysates through ePop autolysis system as well as supernatants containing *E. coli* lysate and secreted proteins from *P. pastoris* were then harvested by centrifugation at 8,000 rpm (g) for 5 min and subsequently incubated for 20 min at 4°C with 1 mL pre-equilibrated Ni-NTA resin (YEASEN *Inc.*), with gentle agitation to allow for binding. Then, they were incubated at room temperature (21°C), treated with a final condition of 4 mM DTT (Dithiothreitol, Sangon Biotech Co., *Ltd.*) and pH 8.5–9.0 for another 16–24 h for protein splicing reaction. The resin was washed three times with 5 mL of washing buffer (20 mM Tris-HCl, 500 mM NaCl, 20 mM imidazole), and proteins were eluted with 10 mL of elution buffer (20 mM Tris-HCl, 500 mM NaCl, 250 mM imidazole). The eluted samples were pooled and dialyzed against ddH_2_O using centrifugal filter units with molecular weight cutoff of 3000 (UFC9003, Millipore). Eluted samples were analyzed by SDS-PAGE, Western blotting with his-tagged antibody and native PAGE analyzed at 560 nm excitation-620 nm emission. For the native PAGE analysis experiment, an SDS-PAGE gel was soaked in Non-SDS running buffer for electrophoresis and further fluorescence detection. Protein stocks were stored at −20°C. All *in situ* splicing assays were performed with three biological replicates. 90 μL overnight cultures, cell lysates as well as purified and concentrated protein samples were added into 384-well microplates for fluorescence detection (excitation: 580nm, emission: 610 nm) unless stated differently (Figures 2B, 3D,F, 4C, 5B,D; Supplemental Figures 1–3).

### *In situ* four strains co-culture for concurrent intein-mediated chimeric protein splicing of two proteins

One cell group was transformed with vectors encoding His-tagged extein^N^-split intein chimeric protein 1 and the ePop autolysis plasmid, while another group expressed His-tagged extein^N^-split intein chimeric protein 2 paired with the same plasmid. Two additional groups were prepared for His-tagged extein^C^-split intein chimeric proteins: one expressing protein 1 and the other protein 2, both combined with the ePop autolysis plasmids. All cultures were incubated overnight at 37°C with shaking at 220 rpm.

Cells harboring vectors for His-tagged C-lobe-split intein chimeric proteins and ePop autolysis plasmids were inoculated into 100 mL M9 medium supplemented with 100 μg/mL kanamycin and 100 μg/mL chloramphenicol at a 1:10 inoculation ratio. Similarly, cells expressing His-tagged N-lobe-split intein chimeric proteins with ePop plasmids were cultured in 100 mL identically supplemented M9 medium using a 1:10 inoculation ratio. For the β-lactamase (Bla)-nitrocefin enzymatic reaction, 20 ng of purified protein samples were combined with 5 mM nitrocefin in a final reaction volume of 100 μL.

For the cross-species co-cultivation for protein splicing experiments, two *P. pastoris* X33 strains were inoculated into 10 mL BMGY medium [10 g/L yeast extract, 20 g/L peptone, 100 mM potassium phosphate, 1.34% yeast nitrogen base (YNB), 4 × 10^−5^% biotin, 1% glycerol] and incubated at 30°C with 220 rpm shaking until reaching mid-log phase (OD_600_ ≈ 6.0). Simultaneously, two *E. coli* BL21 (DE3) cultures were grown in 10 mL LB medium containing 2% glucose, 100 μg/mL kanamycin, and 100 μg/mL chloramphenicol. After 16 h, all four pre-cultures were subcultured at a 1:10 ratio into a 100 mL mixed medium combining 50 mL LB and 50 mL BMMY (identical composition to BMGY except for substitution of glycerol with 0.5% methanol). The co-culture system was maintained at 30°C with 220 rpm agitation for 72 h, with daily methanol supplementation to maintain 0.5% (v/v), ensuring synchronized protein induction across both species.

## Data Availability

The original contributions presented in the study are included in the article/Supplementary Material, further inquiries can be directed to the corresponding authors.
