# Supplementary material for: In situ intein-mediated multiprotein assembly via engineered cross-species consortia
Source: Front Bioeng Biotechnol. 2025 Apr 25;13:1529655. doi: 10.3389/fbioe.2025.1529655 (PMC12062136; doi:10.3389/fbioe.2025.1529655)
Supplement: Supplementary file 2 [file Table1.docx]

**The amino acid sequences of the expressed proteins mentioned in the manuscripts**

1. RGN:

MHHHHHHASSEDVIKEFMRFKVRMEGSVNGHEFEIEGEGEGRPYEGTQTAKLKVTKGGPLPFAWDILSPQFQYGSKAYVKHPADIPDYLKLSFPEGFKWERVMNFEDGGVVTVTQDSSLQDGEFIYKVKLRGTNFPSDGPVMQKKTMGWEASTERMYPEDAGGCFSGDTLVALTDGRSVSFEQLVEEEKQGKQNFCYTIRHDGSIGVEKIINARKTKTNAKVIKVTLDNGESIICTPDHKFMLRDGSYKCAMDLTLDDSLMPLHRKISTTEDSGHA*

His tag

RFPN fragment

Junction sequence

*Ssp* GryB intein N lobe

1. RGC:

MEAVLNYNHRIVNIEAVSETIDVYDIEVPHTHNFALASGVFVHNSAKGALKGEIKMRLKLKDGGHYDAEVKTTYMAKKPVQLPGAYKTDIKLDITSHNEDYTIVEQYERAEGRHSTGAHHHHHH*

His tag

RFPC fragment

Junction sequence

*Ssp* GryB intein C lobe

1. RgN:

MHHHHHHASSEDVIKEFMRFKVRMEGSVNGHEFEIEGEGEGRPYEGTQTAKLKVTKGGPLPFAWDILSPQFQYGSKAYVKHPADIPDYLKLSFPEGFKWERVMNFEDGGVVTVTQDSSLQDGEFIYKVKLRGTNFPSDGPVMQKKTMGWEASTERMYPEDLNRCLSLDTMVVTNGKAIEIRDVKVGDWLESECGPVQVTEVLPIIKQPVFEIVLKSGKKIRVSANHKFPTKDGLKTINSGLKVGDFLRSRAK*

His tag

RFPN fragment

Junction sequence

gp418 intein N lobe

1. RgC:

MCEIFENEIDWDEIASIEYVGVEETIDINVTNDRLFFANGILTHNSAVGALKGEIKMRLKLKDGGHYDAEVKTTYMAKKPVQLPGAYKTDIKLDITSHNEDYTIVEQYERAEGRHSTGAHHHHHH*

His tag

RFPC fragment

Junction sequence

gp418 intein C lobe

1. blaGN:

MHHHHHHMHPETLVKVKDAEDQLGARVGYIELDLNSGKILESFRPEERFPMMSTFKVLLCGAVLSRIDAGQEQLGRRIHYSQNDLVEYSPVTEKHLTDGMTVRELCSAAITMSDNTAANLLLTTIGGPKELTAFLHNMGDHVTRLDRWEPELNEAIPNDERDTTMPVAMATTLRKLLTGAGGCFSGDTLVALTDGRSVSFEQLVEEEKQGKQNFCYTIRHDGSIGVEKIINARKTKTNAKVIKVTLDNGESIICTPDHKFMLRDGSYKCAMDLTLDDSLMPLHRKISTTEDSGHA*

His tag

blaN fragment

Junction sequence

*Ssp* GryB intein N lobe

1. blaGC

MEAVLNYNHRIVNIEAVSETIDVYDIEVPHTHNFALASGVFVHNSAKELLTLASRQQLIDWMEADKVAGPLLRSALPAGWFIADKSGAGERGSRGIIAALGPDGKPSRIVVIYTTGSQATMDERNRQIAEIGASLIKHWHHHHHH*

His tag

blaC fragment

Junction sequence

*Ssp* GryB intein C lobe

1. TetRN-M86N

MSRLDKSKVINSALELLNEVGIEGLTTRKLAQKLGVEQPTLYWHVKNKRALLDALAIEMLDRHHTHFCPLVDACISGDSLISLASTGKRVPIKDLLGEKDFEIWAINEQTMKLESAKVSRVFCTGKKLVYTLKTRLGRTIKATANHRFLTIDGWKRLDELSLKEHIALPRKLEHHHHHH*

His tag

TetRN fragment

Junction sequence

M86 intein N lobe

1. M86C-TetRC

MSSSLQLAPEIEKLPQSDIYWDPIVSITETGVEEVFDLTVPGLRNFVANDIIVHNSDLEGESWQDFLRNNAKSFRCALLSHRDGAKVHLGTRPTEKQYETLENQLAFLCQQGFSLENALYALSAVGHFTLGCVLEDQEHQVAKEERETPTTDSMPPLLRQAIELFDHQGAEPAFLFGLELIICGLEKQLKCESGSHHHHHH*

His tag

TetRC fragment

Junction sequence

M86 intein C lobe
