# Supplementary material for: In situ intein-mediated multiprotein assembly via engineered cross-species consortia
Source: Front Bioeng Biotechnol. 2025 Apr 25;13:1529655. doi: 10.3389/fbioe.2025.1529655 (PMC12062136; doi:10.3389/fbioe.2025.1529655)
Supplement: Supplementary file 3 [file DataSheet1.pdf]

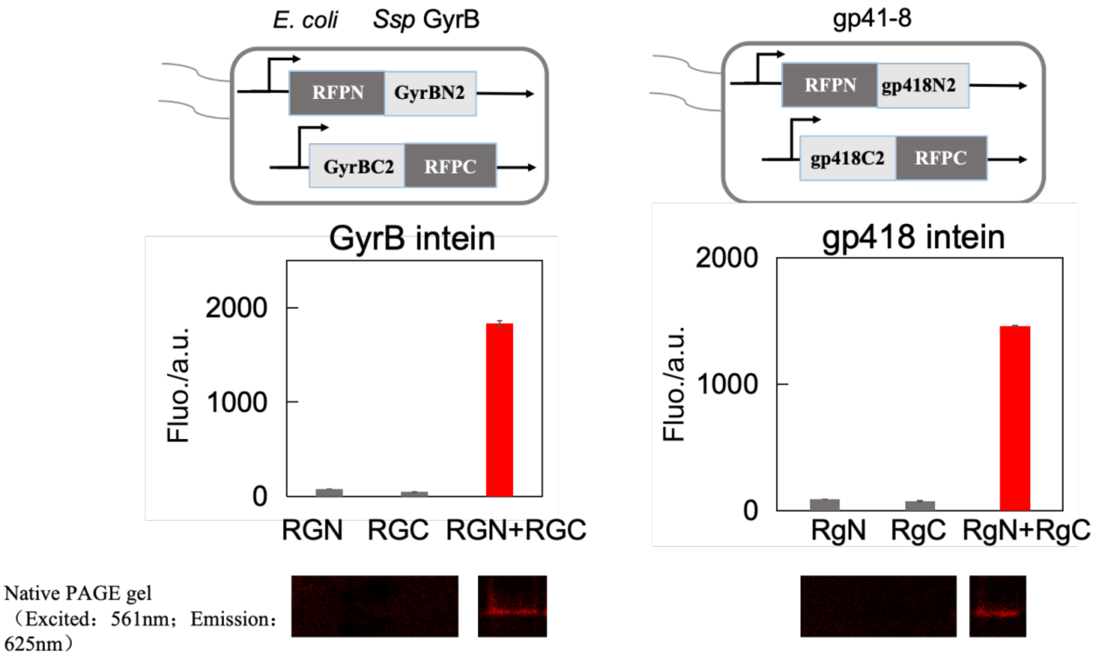

Supplemental information figure 1 Split intein splicing *in vivo*. Characterization of the splicing efficiency of *Ssp* GyrB and gp418 was first examined in *E. coli* by co-transforming two plasmids expressing extein^{N}-GyrB^{N}-, GyrB^{C}-extein^{C} chimeric proteins and extein^{N}-gp418^{N}-, gp418^{C}-extein^{C}. respectively.

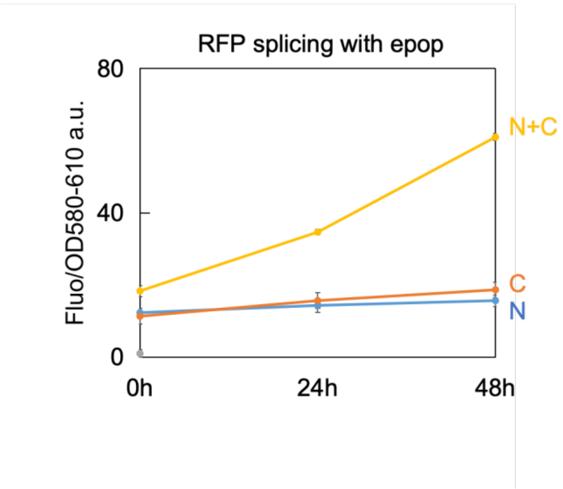

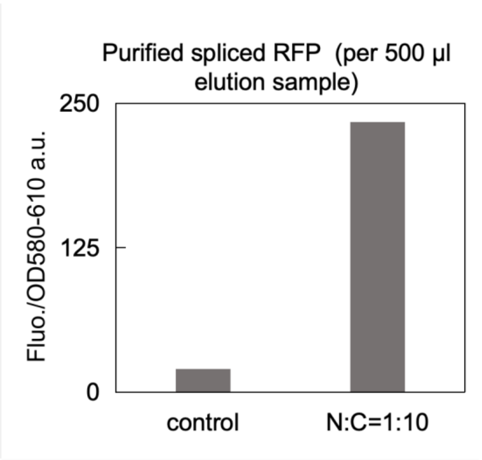

Supplemental information figure 2 Auto-lysis consortia enable mCherry assembly. 90 μl overnight cultures were added into 384-well microplates for fluorescence detection (excitation: 580nm, emission: 610nm).

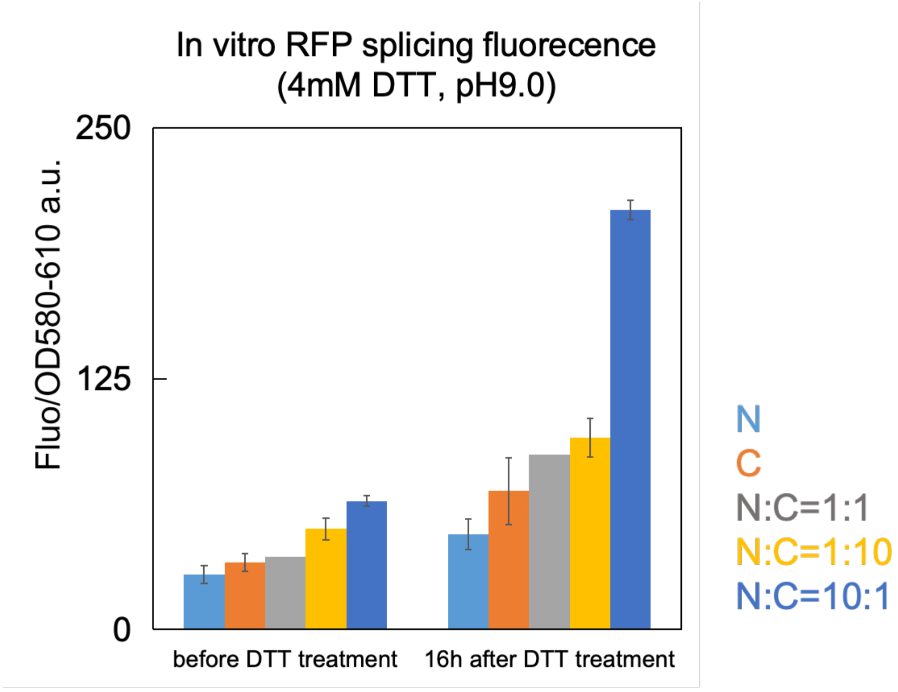

Supplemental information figure 3 Optimizing intein splicing reaction by 4 mM DTT treatment. Overnight cultures were incubated at room temperature (21°C), treated with a final condition of 4 mM DTT (Sangon Biotech Co., Ltd.) and pH 8.5-9.0 for another 16-24 h for protein splicing reaction. 90 μl overnight cultures were added into 384-well microplates for fluorescence detection (excitation: 580nm, emission: 610nm).

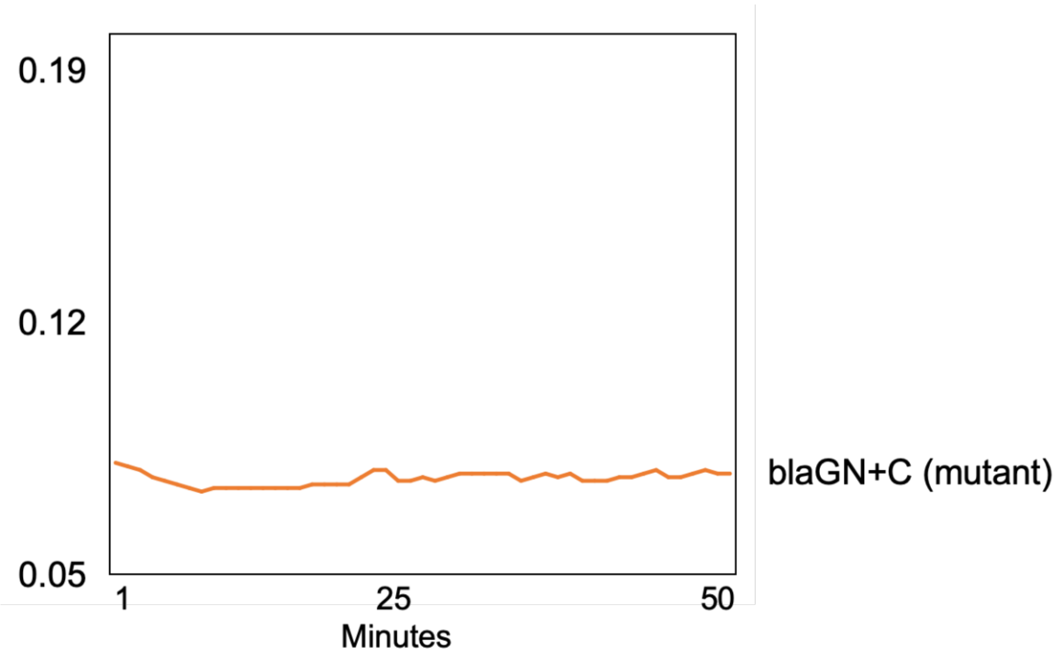

Supplemental information figure 4 Bla negative control. A negative control showing the β-Lactamase mutant cannot react with nitrocefin substrates to increase absorbance values at OD490nm, showing the mutation on β-Lactamase completely block protein splicing and inhibit enzymatic ability.

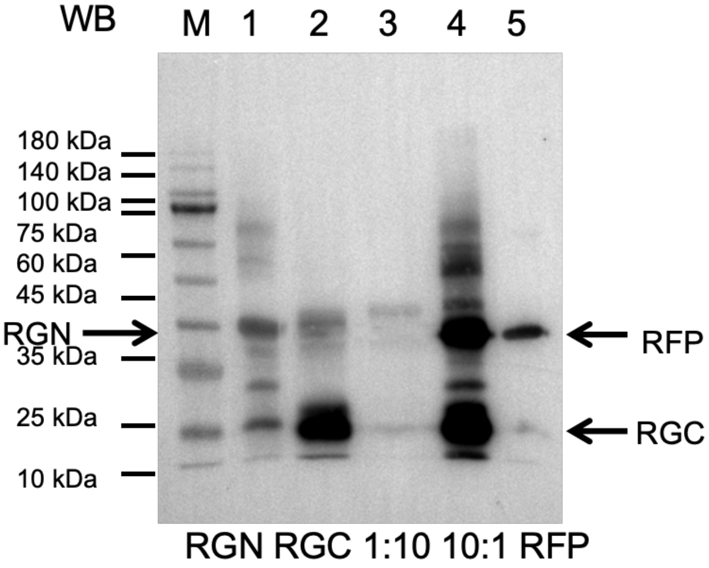

Supplemental information figure 5 Demonstration of *in situ* protein splicing by *E. coli* culture to assemble mCherry (original data). Western blots analysis results of eluted samples of extein^{N}-GyrB^{N}- as well as GyrB^{C}-extein^{C} chimeric proteins, spliced RFP (mCherry) and RFP positive

control. 1:10: the subculture ratio of the strain expressing extein^{N}-GyrB^{N}- and the other one expressing GyrB^{C}-extein^{C} follows a 1:10 ratio. 10:1: the subculture ratio of the strain expressing extein^{N}-GyrB^{N}- and the other one expressing GyrB^{C}-extein^{C} follows a 10:1 ratio.
